# Pulmonary shunt in critical care: a practical approach with clinical scenarios

**DOI:** 10.1186/s44158-024-00147-5

**Published:** 2024-03-06

**Authors:** Davide Raimondi Cominesi, Mario Forcione, Matteo Pozzi, Marco Giani, Giuseppe Foti, Emanuele Rezoagli, Francesco Cipulli

**Affiliations:** 1 School of Medicine and Surgery, University of Milano-Bicocca, Monza, Italy; 2 Department of Emergency and Intensive Care, Fondazione IRCCS San Gerardo Dei Tintori, Monza, Italy

**Keywords:** Pulmonary shunt, ARDS, Oxygenation, Perfusion, ECMO

## Abstract

**Background:**

Pulmonary shunt refers to the passage of venous blood into the arterial blood system bypassing the alveoli-blood gas exchange. Pulmonary shunt is defined by a drop in the physiologic coupling of lung ventilation and lung perfusion. This may consequently lead to respiratory failure.

**Main body:**

The pulmonary shunt assessment is often neglected. From a mathematical point of view, pulmonary shunt can be assessed by estimating the degree of mixing between oxygenated and deoxygenated blood. To compute the shunt, three key components are analyzed: the oxygen (O_2_) content in the central venous blood before gas exchange, the calculated O_2_ content in the pulmonary capillaries after gas exchange, and the O_2_ content in the arterial system, after the mixing of shunted and non-shunted blood. Computing the pulmonary shunt becomes of further importance in patients on extracorporeal membrane oxygenation (ECMO), as arterial oxygen levels may not directly reflect the gas exchange of the native lung.

**Conclusion:**

In this review, the shunt analysis and its practical clinical applications in different scenarios are discussed by using an online shunt simulator.

**Graphical Abstract:**

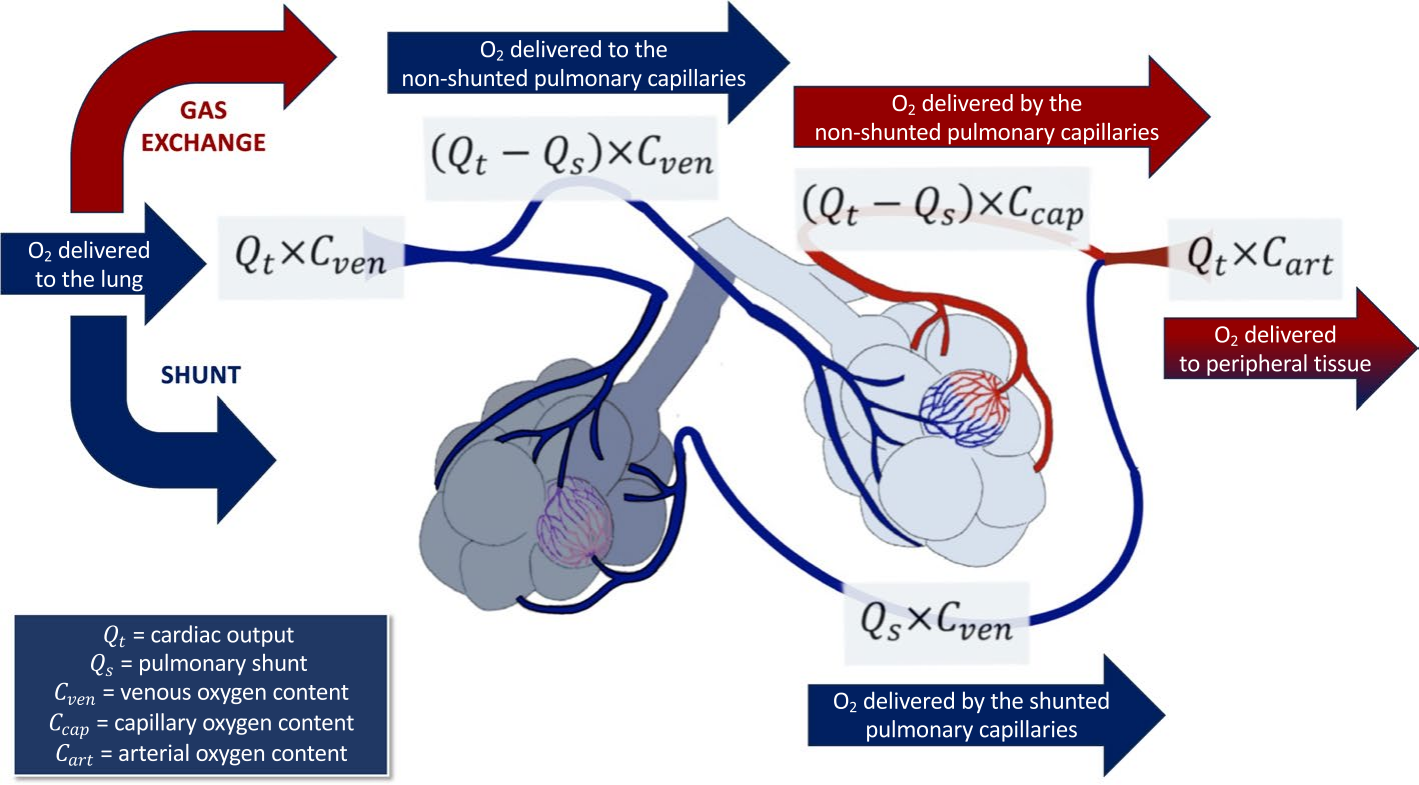

## Background

The pulmonary shunt is defined as the passage of deoxygenated blood into the arterial blood system, thereby bypassing (functionally or anatomically) the gas exchange at the alveolo-capillary level [1]. The degree of the pulmonary shunt is related to the severity of hypoxemia, and its changes correlate with the course of the disease [2].

The assessment of gas exchange is usually limited to the arterial blood gas analysis (BGA), whereas the measurement of pulmonary shunt is not often considered as standard practice [3]. However, the arterial oxygen content can be markedly influenced by variations of central venous blood saturation, which may occur in specific clinical scenarios. For instance, low cardiac output may markedly reduce SvO_2_, whereas patients on extracorporeal membrane oxygenation (ECMO) show very high venous oxygen content [4]. Pulmonary shunt quantification and interpretation could facilitate the assessment of the disease severity, the continuous monitoring of the evolution of the pulmonary impairment, and monitoring the trajectory of the response to treatments [5, 6].

In this review, starting from the physic concepts expressed by Gay-Lussac in 1802 in the manuscript entitled “Researches on the expansion of gases and vapors” [7], passing through the physiological works of West and Riley [5, 8] delivered in the second part of the past century and finally arriving to the most recent proposed mathematical models and calculators [9], the pulmonary shunt computation is comprehensively explored by reviewing the meaning of each equation variable.

Additionally, we provided some clinical examples that were specifically thought to underline how this analysis may be useful for clinicians in dealing with different cardiorespiratory failure scenarios, taking advantage of an advanced online shunt simulator.

The discussion of the complex effect of shunt on CO_2_ elimination is beyond the scope of the current work; therefore, it will not here be described.

## Definitions: shunt, ventilation/perfusion mismatch, and dead space

Shunt, ventilation-perfusion mismatch (V/Q), and dead space refer to specific abnormalities of lung ventilation and perfusion.

Shunt, as explained above, represents the scenario where some regions of the lungs receive blood flow but no ventilation, resulting in a decrease in oxygen levels in the blood. The presence of shunt can be observed in ARDS patients, who typically exhibit extensive consolidations on computed tomography (CT) scan. From a pathophysiological perspective, there is a notable reduction in functional residual capacity (FRC); this reduction can be effectively treated with PEEP to restore end-expiratory lung volume (EELV) [10, 11].

V/Q mismatch is an imbalance between ventilation and blood flow. This ratio can be low (shunt-like) or high (dead space-like). Shunt-like mismatch results from reduced ventilation relative to blood flow, leading to decreased oxygenation. Dead space-like mismatch occurs when ventilation exceeds blood flow, leading to inadequate carbon dioxide removal.

Dead space refers to the regions of the respiratory system which do not participate to gas exchange. Total dead space (also known as physiological dead space) is the sum of the dead space of the airways and alveolar dead space. While the former is physiological, the latter occurs when alveoli are ventilated but not perfused due to pathological conditions, such as pulmonary embolism.

In summary, shunt, V/Q mismatch, and dead space are interrelated concepts that describe different aspects of impaired gas exchange in the lungs. Understanding these principles is crucial for the diagnosis and the management of respiratory disorders [12].

The current review mainly focuses on the use of shunt calculation and its useful applications at bedside in different critical care scenarios.

## Oxygen blood content

The O_2_ blood content (${C}_{{O}_{2}}$) can be defined as the volume of O_2_ contained in each deciliter of blood (O_2_ mL/dL of blood). In the bloodstream, O_2_ is largely bound to hemoglobin and dissolved only in a small part. The O_2_ quantity contained in blood is proportional to the hemoglobin level and its O_2_ saturation, and to the O_2_ partial pressure (${P}_{{O}_{2}}$), as expressed in the following equation:

1 $${C}_{{O}_{2}}=X \times Hb \times \frac{{S}_{{O}_{2}}}{100} +\mathrm{Y }\times {P}_{{O}_{2}}$$

where $Hb$ is the hemoglobin concentration expressed as grams per deciliter, $\frac{{S}_{{O}_{2}}}{100}$ is the hemoglobin saturation expressed as a ratio, and *X* and *Y* are coefficients respectively for the O_2_ bound by a gram of hemoglobin and the O_2_ solubility coefficient.

According to Charles’ law for isobaric (i.e., constant pressure) transformation, the volume occupied by a gas can be computed as follows [7, 13]:

2 $$V = \frac{nRT}{P}$$

where the dependent variable $V$ is the oxygen volume released by the total dissociation of 1 g of hemoglobin, $n$ is the gas quantity (${\text{mol}}$), $P$ is the atmospheric pressure (1 ${\text{atm}}$), $T$ is the absolute temperature (at 37 °C is equal to 310.15 °K), and $R$ is the universal gas constant ($8.21\times {10}^{-5} {{\text{m}}}^{3}\mathrm{ atm}{\mathrm{ K}}^{-1}{\mathrm{ mol}}^{-1}$).

One mole of fully O_2_-bound hemoglobin contains four moles of oxygen; 1 g of hemoglobin is equal to $1.471\times {10}^{-5}$ moles (mass 68,000 KDa). It can be derived that 1 g of hemoglobin yields $5.882\times {10}^{-5}$ moles of oxygen ($n$).

Substituting these values into Eq. 2, the amount of oxygen volume released by a gram of hemoglobin after complete dissociation can be calculated as follows:

3 $$V = \frac{5.882\times {10}^{-5}\times 8.21\times {10}^{-5} \times 310.15}{1} =1.50\times {10}^{-6} {{\text{m}}}^{3}=1.497 ml$$

Oxygen is not an ideal gas. Consequently, a correcting factor (*Z* = 0.91) is necessary to account for the gas’ compressibility [14, 15].

The actual volume of oxygen released by the dissociation of 1 g of hemoglobin is then as follows:

4 $$V \times \mathrm{Z }=1.50 \times 0.91 =1.363 ml$$

At sea level, while atmospheric pressure is constant, the human body temperature can range from 28 °C (301.15 °K, severe hypothermia) to 42°C (315.15 °K, severe hyperthermia). The patient’s temperature must be considered for a precise oxygen volume computation (Table 1).

**Table 1:** Oxygen volume released through the total dissociation of 1 g of hemoglobin as a function of body temperature

Temperature (°C)	Temperature (°K)	Pressure (atm)	O_2_ volume (ml)
28	301.15	1	1.323
34	307.15	1	1.350
38	311.15	1	1.367
42	315.15	1	1.385

Based on Eq. 1, ${C}_{{O}_{2}}$ can be finally computed as follows:

5 $${C}_{{O}_{2}}=\left(1.363 \times Hb \times \frac{{So}_{2}}{100} \right)+ 0.003 \times {P}_{{O}_{2}}$$

Based on Table 1, the volume of oxygen released by the dissociation of a gram of hemoglobin at 37 °C is 1.363 ml. In case of fever, it can increase up to 1.385 ml, or, to the contrary, in case of severe hypothermia, it can drop to 1.323 ml. For example, in a patient with 10 g/dl of $Hb$, 80 mmHg of ${P}_{{O}_{2}}$, and 99% of ${So}_{2}$, the increase in ${C}_{{O}_{2}}$ from 37 to 40 °C is 0.2 ml/dl.

It is worth to underline that in the ${So}_{2}$ computation, the presence of methemoglobin (MetHb) and carboxyhemoglobin (COHb) must be considered. Most blood gas analyzer report the hemoglobin O_2_ saturation after accounting for methemoglobin and carboxyhemoglobin as “HbO_2_.” When available, HbO_2_ should be preferred to ${So}_{2}$ (Eq. 5 above).

## Pulmonary shunt

**Fig. 1:**
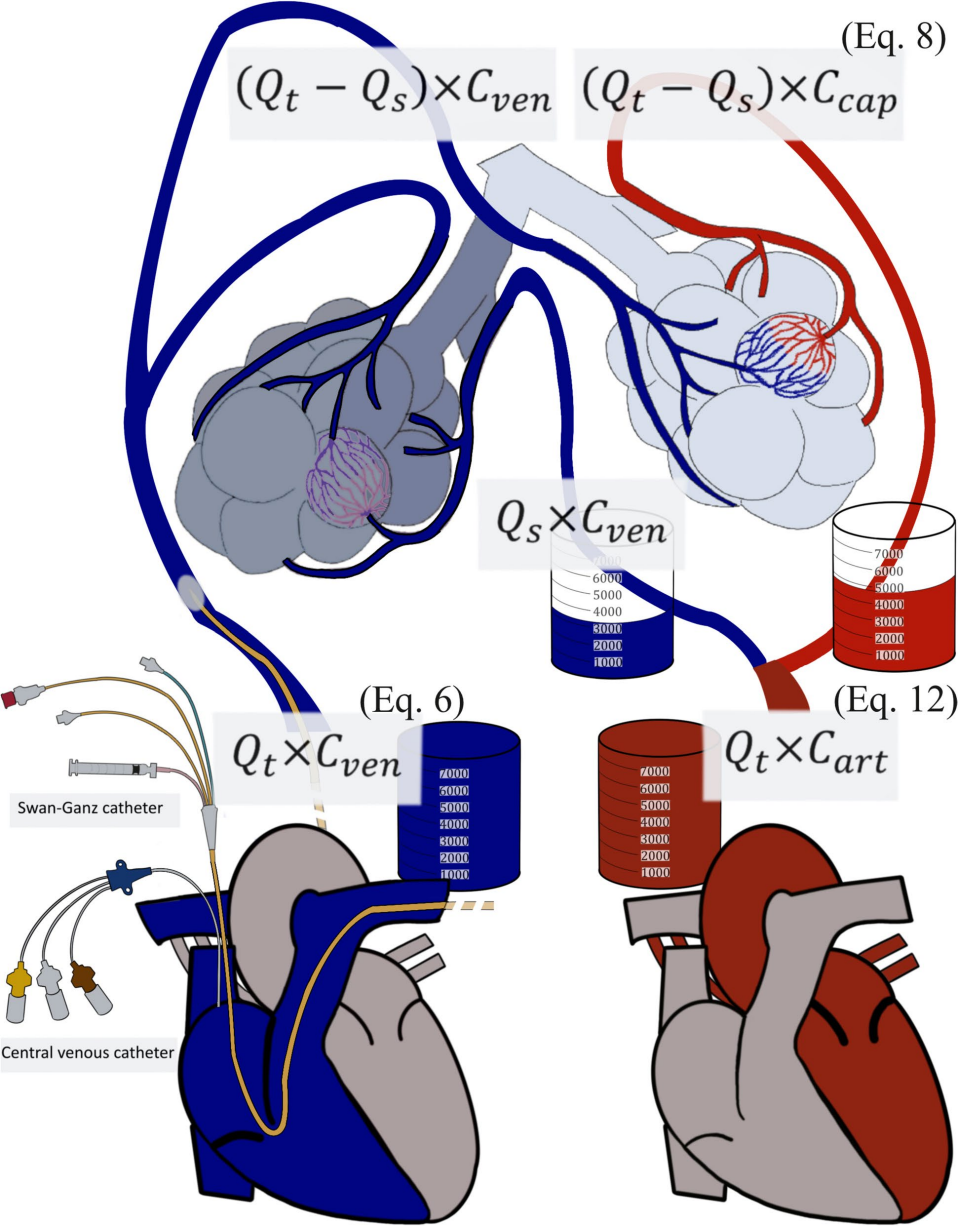
Illustration of the sampling sites required for pulmonary shunt calculation. The Swan-Ganz catheter allows blood sampling in the pulmonary artery, enabling the measurement of mixed venous O_2_ saturation (${{\varvec{S}}}_{{\varvec{v}}{{\varvec{O}}}_{2}}$), whereas the central venous catheter samples blood from the superior vena cava — right atrium junction, enabling the measurement of the so-called central venous O_2_ saturation (${{\varvec{S}}}_{{\varvec{c}}{\varvec{v}}{{\varvec{O}}}_{2}}$). Displayed formulas are discussed in the text. ${{\varvec{C}}}_{{\varvec{a}}{\varvec{r}}{\varvec{t}}}$, arterial O_2_ content; ${{\varvec{C}}}_{{\varvec{c}}{\varvec{a}}{\varvec{p}}}$, capillary O_2_ content; ${{\varvec{C}}}_{{\varvec{v}}{\varvec{e}}{\varvec{n}}}$, venous O_2_ content; ${{\varvec{Q}}}_{{\varvec{s}}}$, pulmonary shunt; ${{\varvec{Q}}}_{{\varvec{t}}}$, total amount of blood transported to the lungs

The pulmonary shunt computation is based on the analysis of ${C}_{{O}_{2}}$ at three sites [16, 17]: arterialized blood in the lung capillaries after the gas exchange (${C}_{cap}$); venous blood from the pulmonary arteries (${C}_{ven}$); and arterial blood (${C}_{art}$) (Fig. 1) [8, 18].

### Venous oxygen content

In each minute, a given quantity of venous blood is pumped from the right side of the heart to the pulmonary circulation (${Q}_{t}$) carrying a given amount of oxygen (${C}_{ven}$). The oxygen delivered to the lung can be computed as follows:

6 $${Q}_{t}\times {C}_{ven}$$

Two sites can be used to get the BGA sample for the ${C}_{ven}$ computation: the right atrium via the central venous line or the pulmonary artery via the Swan-Ganz catheter (Fig. 1). The oxygen content from these two sites is often similar, although the O_2_ content in the pulmonary artery is usually slightly lower than that in the right atrium [19, 20] due to the composition of the blood brought to the heart from the coronary sinus and the anterior cardiac veins, which is very low in oxygen. Studies showed that ${S}_{cv{O}_{2}}$ can be used as a reliable and less risky alternative to estimate ${S}_{v{O}_{2}}$ while still maintaining high accuracy [21–23]. Values obtained via the Swan-Ganz catheter may be more accurate than those sampled via the central venous catheter, as the latter may be sampled too proximal as in the superior vena cava (higher oxygen content from the superior part of the body) or sampled too distal near the coronary sinus outlet (lower oxygen content from the heart). Using both the central line and the Swan-Ganz catheter is strongly recommended to refer to the same sampling site when multiple recordings are performed on the same patient.

Based on Eq. 5, the ${C}_{ven}$ measurement follows the equation:

7 $${C}_{ven}=\left(1.363\times Hb\times \frac{{S}_{v{O}_{2}}}{100}\right)+ 0.003 \times {P}_{v{O}_{2}}$$

where ${S}_{v{O}_{2}}$ is the hemoglobin O_2_ saturation and ${P}_{v{O}_{2}}$ is the O_2_ partial pressure in the right atrium and/or pulmonary arteries.

### Capillary oxygen content

The non-shunted blood flow is calculated by subtracting the shunt fraction ($${Q}_{s})$$ from the cardiac output ($${Q}_{t}$$) [1]. The total amount of oxygen transported within the pulmonary capillaries is determined by multiplying $${C}_{cap}$$ by the non-shunted blood flow (Fig. 1):

8 $$\left({Q}_{t}-{Q}_{s}\right)\times {C}_{cap}$$

where ${C}_{cap}$ is the O_2_ concentration in the pulmonary capillaries after the blood-gas exchange prior to reaching the left heart and the systemic circulation.

**Fig. 2:**
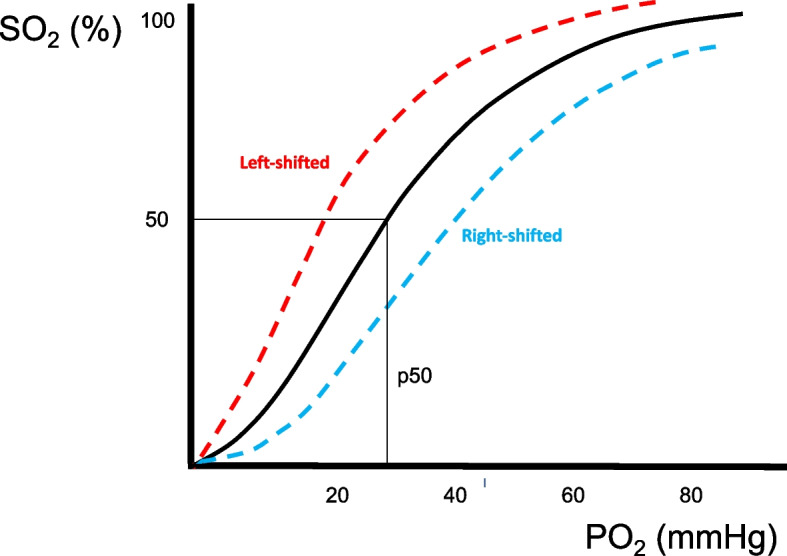
Oxygen-hemoglobin dissociation curve. Of note, conditions that shift the curve to the left (dashed red line: high pH, low temperature, low 2,3-diphosphoglicerate concentration) increase the oxygen affinity and the hemoglobin saturation, while conditions that shift the curve to the right (dashed light blue line: low pH, high temperature, high 2,3-diphosphoglicerate concentration) decrease the oxygen affinity and the hemoglobin saturation. p50, pressure at which hemoglobin is 50% saturated (27 mmHg on the *X*-axis within a normal representative oxygen-hemoglobin dissociation curve) [24]. On the *Y*-axis, we reported oxygen saturation expressed as %. On the *X*-axis, we reported arterial oxygen pressure expressed as mmHg

Sampling the blood in these capillaries is clearly not possible. Thus, the O_2_ saturation in the capillaries (${S}_{cap{O}_{2}}$) should be derived from the hemoglobin dissociation curve knowing the capillary O_2_ partial pressure (Fig. 2).

Assuming a state of equilibrium where the alveolar-blood oxygen gradient is null, the O_2_ partial pressure in the capillaries (${P}_{cap{O}_{2}}$) is equal to the alveolar O_2_ partial pressure:

9 $${P}_{cap{O}_{2}}=\left(\left({P}_{atm}- {P}_{{H}_{2}O}\right) \times {F}_{i}{O}_{2}\right)-\frac{{P}_{a}{CO}_{2}}{RQ}$$

where ${P}_{atm}$ is the atmospheric pressure (at sea level 760 mmHg), ${P}_{{H}_{2}O}$ is the vapor pressure (reference value 47 mmHg), ${F}_{i}{O}_{2}$ is the inspiratory O_2_ fraction, ${P}_{a}{CO}_{2}$ is the arterial partial CO_2_ pressure, and $RQ$ is the respiratory quotient. The latter is a dimensionless number which ranges from 0.7 to 1.2, defined as the ratio between the volume of CO_2_ (VCO_2_) produced and volume of O_2_ consumed per minute. $RQ$ is a valuable parameter used in respiratory physiology to assess what kind of substrate has been used for metabolism [25] and estimate the alveolar CO_2_ partial pressure. Of note, in a clinical scenario in which VO_2_, VCO_2_, and consequently RQ are not disposable, it is possible to assume RQ equal to 0.8 [26].

Based on the assumptions on ${P}_{cap{O}_{2}}$, Eq. 5 can be adapted to measure ${C}_{cap}$:

10 $${C}_{cap}=\left(1.363\times Hb\times \frac{{S}_{cap{O}_{2}}}{100}\right)+ 0.003 \times {P}_{cap{O}_{2}}$$

As introduced above, ${S}_{cap{O}_{2}}$ cannot be measured directly but must be derived using the following equation:

11 $${S}_{cap{O}_{2}}= 100\mathrm{\%}-\left(\mathrm{\%COHb}+\mathrm{\%MetHb}\right)$$

### Arterial oxygen content

Each minute, a given quantity of blood is pumped by the left ventricle to the peripheric tissues carrying a given quantity of oxygen ${C}_{art}$ (Fig. 1). The oxygen delivered to the peripheral tissues can be calculated as follows:

12 $${Q}_{t}\times {C}_{art}$$

Based on Eq. 5, the computation of ${C}_{art}$ can be expressed as follows:

13 $${C}_{art}=\left(1.363\times Hb\times \frac{{S}_{a{O}_{2}}}{100}\right)+ 0.003 \times {P}_{{aO}_{2}}$$

### Pulmonary shunt computation

The shunt fraction is calculated as the ratio of the blood flow that is not subject to gas exchange (${Q}_{s}$) to the cardiac output (${Q}_{t}$) [27–30]:

14 $$\frac{{Q}_{s}}{{Q}_{t}}$$

${C}_{art}$ results from mixing of oxygenated and non-oxygenated blood (from the shunted capillaries), considering their relative blood flows:

15 $$\left({Q}_{t}\times {C}_{art}\right)=\left({Q}_{s} \times {C}_{ven}\right)+ \left[\left({Q}_{t}-{Q}_{s}\right) \times {C}_{cap}\right]$$

Expanding Eq. 15:

16 $$\left({Q}_{t}\times {C}_{art}\right)=\left({Q}_{s} \times {C}_{ven}\right)+({Q}_{t} \times {C}_{cap})-({Q}_{s}\times {C}_{cap})$$

17 $$\left({Q}_{t}\times {C}_{art}\right)-({Q}_{t} \times {C}_{cap})=\left({Q}_{s} \times {C}_{ven}\right)-({Q}_{s}\times {C}_{cap})$$

18 $$\left({Q}_{t}\times {C}_{cap}\right)-({Q}_{t} \times {C}_{art})=({Q}_{s}\times {C}_{cap})-\left({Q}_{s} \times {C}_{ven}\right)$$

Factorizing and rearranging:

19 $${Q}_{t}\times \left({C}_{cap}-{C}_{art}\right)={Q}_{s}\times \left({C}_{cap}-{C}_{ven}\right)$$

Bringing to the final formula for shunt calculation:

20 $$\frac{{Q}_{s}}{{Q}_{t}}=\frac{{C}_{cap}-{C}_{art}}{{C}_{cap}-{C}_{ven}}$$

Expressing the O_2_ blood contents reported in Eq. 19, we finally get the following:

21 $$\frac{Qs}{Qt}=\frac{\left[\left(1.363\times {\text{Hb}}\times {S}_{cap{O}_{2}}\right)+\left(0.003\times {P}_{cap{O}_{2}}\right)\right]-[\left(1.363\times {\text{Hb}}\times {S}_{a{O}_{2}}\right)+\left(0.003\times {P}_{a{O}_{2}}\right)] }{[\left(1.363\times {\text{Hb}}\times {S}_{cap{O}_{2}}\right)+\left(0.003\times {P}_{cap{O}_{2}}\right)]-[(1.363\times {\text{Hb}}\times {S}_{v{O}_{2}})+(0.003\times {P}_{v{O}_{2}})]}$$

**Fig. 3:**
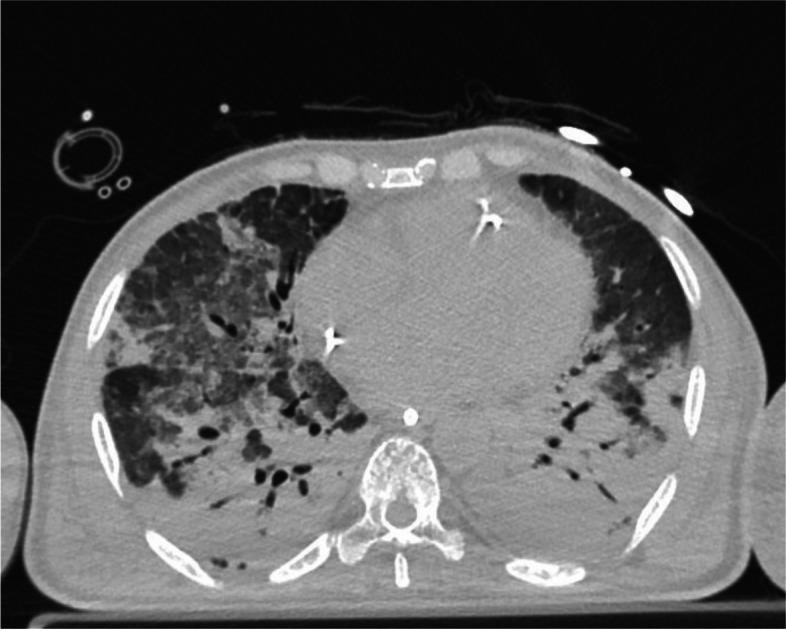
CT scan of an ARDS patient on V-V ECMO with a high shunt. In this figure, we report an exemplary lung CT image of a patient undergoing V-V ECMO for severe respiratory failure with a shunt of 69%. It is visible the massive consolidation of the basal bilateral lung regions and the ground-glass opacities of the middle and upper lung areas

The arterial oxygen content/saturation relies on two factors. Firstly, it is affected by the oxygen saturation of the venous blood that perfuses the lungs and, secondly, by the efficiency of the lungs in gas exchange (shunt fraction). By keeping constant the shunt fraction, it can be confirmed that the arterial oxygen saturation correlates linearly with the venous oxygen saturation. In instances of low cardiac output syndrome, the venous oxygen saturation decreases due to heightened oxygen extraction, consequently leading to a decline in arterial oxygen saturation. Conversely, in patients treated with veno-venous (V-V) ECMO, the venous oxygen saturation notably rises due to the artificial oxygenation of venous blood facilitated by the membrane lung oxygenation. Consequently, there is an increase in arterial oxygen saturation, even in the presence of significant lung damage (Fig. 3). In both scenarios, the arterial oxygen saturation does not accurately represent the severity of respiratory failure. In certain cases, understanding the severity of respiratory failure depends more on calculating the shunt fraction rather than analyzing arterial oxygen saturation.

## Application of pulmonary shunt measurement in clinical practice

In this section, different clinical scenarios are described wherein the measurement of the pulmonary shunt aids the patient’s assessment [9].

It should be noted that any interpretation of the pulmonary shunt should be limited to the assessment of the type of gas-exchange impairments, without inferring their specific etiology.

### Patient 1: Acute respiratory distress syndrome (ARDS)

A 55-year-old male is admitted to ICU due to primary ARDS caused by H_1_N_1_ influenza. The patient is sedated and mechanically ventilated under volume-controlled ventilation.

The arterial BGA shows severe hypoxic respiratory failure (Table 2). The patient is hemodynamically stable without vasopressors. The venous BGA shows a normal ${S}_{{vO}_{2}}$: 68.4%. To estimate the severity of the respiratory failure, the pulmonary shunt can be computed using Eq. 21 and results 0.4 (40%).

**Table 2:** Baseline respiratory and hemodynamic parameters

Parameters	Values
Tidal volume (ml)	380
Respiratory rate (breaths/min)	24
PEEP (cmH_2_O)	12
FiO_2_ (%)	60
Plateau pressure (cmH_2_O)	24
Driving pressure (cmH_2_O)	12
Respiratory system compliance (ml/cmH_2_O)	31.7
pH	7.4
P_a_O_2_ (mmHg)	62
P_a_CO_2_ (mmHg)	40
HbO_2_ (%)	90
P_v_CO_2_ (mmHg)	45
P/F (mmHg)	103
COHb (%)	1
MetHb (%)	0.6
PA (mmHg)	120/80
HR (beats/min)	85
Qt (L/min)	8
SV (ml)	94.2
Body temperature (°C)	37

*COHb*, Carboxyhemoglobin, *FiO*_*2*_ Fraction of inspired oxygen, *HbO*_*2*_ Hemoglobin oxygen saturation in arterial blood, *HR *Heart rate, *MetHb *Methemoglobin, *PA *Arterial blood pressure, *P*_*a*_*CO*_*2*_ Partial pressure of carbon dioxide in arterial blood, *P*_*a*_*O*_*2*_ Partial pressure of oxygen in arterial blood, *PEEP *Positive end-expiratory pressure, *P/F*, P_a_O_2_/ FiO_2_ ratio; *P*_*v*_*CO*_*2*_, partial pressure of carbon dioxide in venous blood, *Qt *Cardiac output, *SV *Stroke volume

**Fig. 4:**
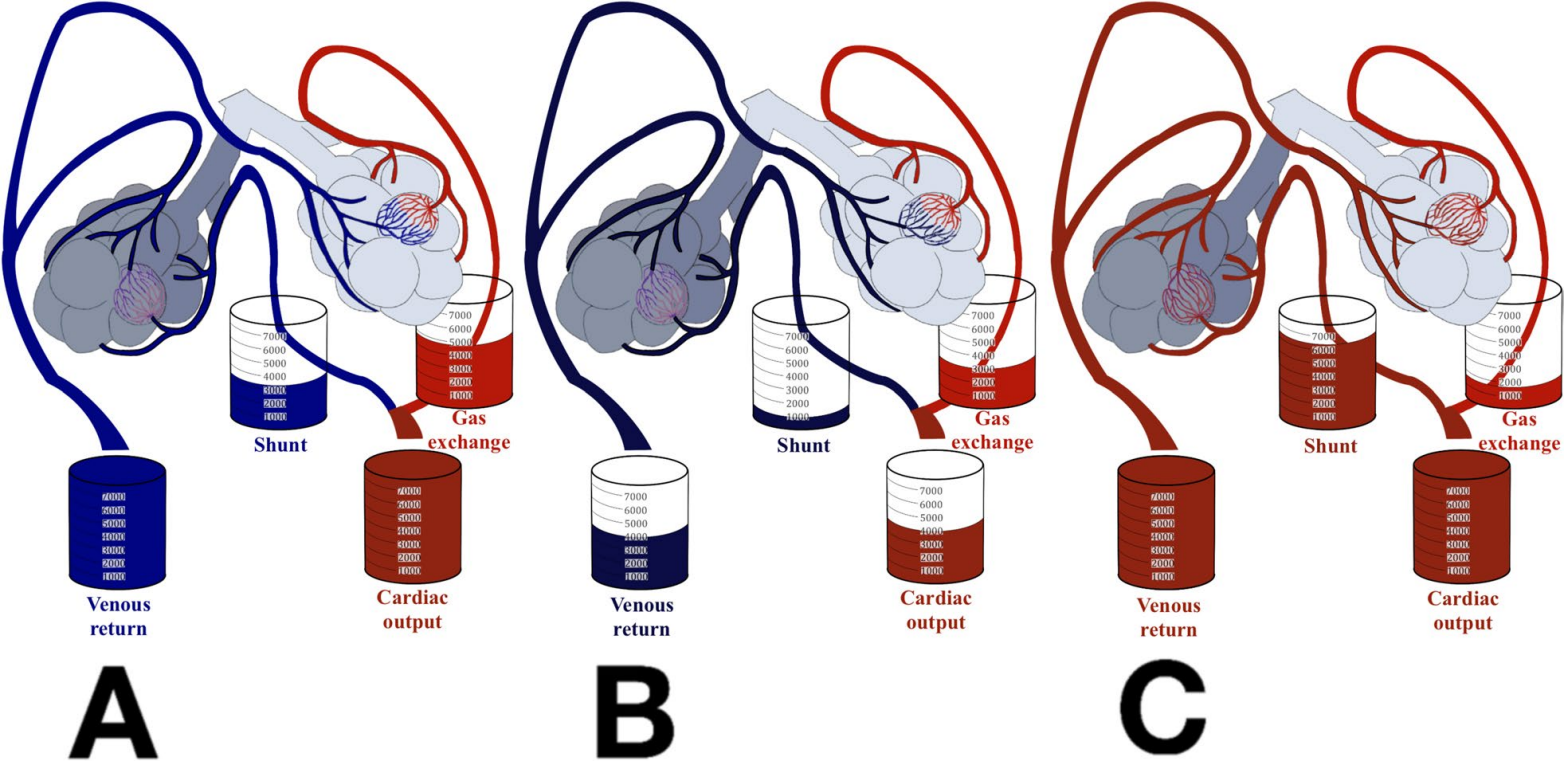
Illustration of three distinct clinical scenarios involving pulmonary shunt. **A** Patient 1 — ARDS. **B** Patient 2 — lobar pneumonia with reduced cardiac output. **C** Patient 3 — ARDS on V-V ECMO

In this case, the high degree of shunt is combined with a low respiratory system compliance, which is common in ARDS patients [31] (Fig. 4A).

### Patient 2: Lobar pneumonia

A 75-year-old male is admitted to ICU due to lobar pneumonia. The patient is sedated and mechanically ventilated under volume-controlled ventilation (Table 3).

**Table 3:** Baseline respiratory and hemodynamic parameters

Parameters	Values
Tidal volume (ml)	400
Respiratory rate (breaths/min)	18
PEEP (cmH_2_O)	10
FiO_2_ (%)	60
Plateau pressure (cmH_2_O)	18
Driving pressure (cmH_2_O)	8
Respiratory system compliance (ml/cmH_2_O)	50
pH	7.4
P_a_O_2_ (mmHg)	62
P_a_CO_2_ (mmHg)	40
HbO_2_ (%)	90
P_v_CO_2_ (mmHg)	50
P/F (mmHg)	103
COHb (%)	1
MetHb (%)	0.6
PA (mmHg)	125/75
HR (beats/min)	90
Qt (L/min)	4
SV (ml)	44.4
Body temperature (°C)	37

*COHb *Carboxyhemoglobin, *FiO*_*2*_ Fraction of inspired oxygen, *HbO*_*2*_ Hemoglobin oxygen saturation in arterial blood, *HR *Heart rate, *MetHb *Methemoglobin, *PA *Arterial blood pressure, *P*_*a*_*CO*_*2*_ Partial pressure of carbon dioxide in arterial blood, *P*_*a*_*O*_*2*_ Partial pressure of oxygen in arterial blood, *PEEP *Positive end-expiratory pressure, *P/F*, P_a_O_2_/FiO_2_ ratio; *P*_*v*_*CO*_*2*_ Partial pressure of carbon dioxide in venous blood, *Qt *Cardiac output, *SV *Stroke volume

The arterial BGA shows severe hypoxia (Table 3). The venous BGA shows a ${S}_{{vO}_{2}}$ of 46.1%, suggesting a condition of low-cardiac output. To estimate the severity of the respiratory failure, the pulmonary shunt is measured to be 0.25 (25%).

Compared to the first patient, pulmonary shunt is lower, as the disease is limited to a single area of the lung, but the low central venous blood saturation results in the same degree of arterial hypoxemia (Fig. 4B).

### Patient 3: ARDS on V-V ECMO

A 45-year-old female is admitted to ICU due to primary ARDS caused by H_1_N_1_ influenza and supported by V-V ECMO. The patient is sedated and mechanically ventilated under volume-controlled ventilation (Table 4).

**Table 4:** Baseline respiratory and hemodynamic and ECMO parameters

Parameters	Values
Blood flow (L/min)	4
Gas flow (L/min)	4
FiO_2_ ML (%)	60
Tidal volume (ml)	320
Respiratory rate (breaths/min)	10
PEEP (cmH_2_O)	14
FiO_2_ NL (%)	60
Plateau pressure (cmH_2_O)	24
Driving pressure (cmH_2_O)	10
Respiratory system compliance (ml/cmH_2_O)	32
pH	7.4
P_a_O_2_ (mmHg)	62
P_a_CO_2_ (mmHg)	40
HbO_2_ (%)	90
P_v_CO_2_ (mmHg)	42
COHb (%)	1
MetHb (%)	0.6
PA (mmHg)	120/80
HR (beats/min)	90
Qt (L/min)	8
SV (ml)	88.9
Body temperature (°C)	37

*COHb *Carboxyhemoglobin, *FiO*_*2*_ Fraction of inspired oxygen, *HbO*_*2*_ Hemoglobin oxygen saturation in arterial blood, *HR *Heart rate, *MetHb *Methemoglobin, *ML *Membrane lung, *NL *Natural lung, *PA *Arterial blood pressure, *P*_*a*_*CO*_*2*_, partial pressure of carbon dioxide in arterial blood; *P*_*a*_*O*_*2*_ Partial pressure of oxygen in arterial blood, *PEEP *Positive end-expiratory pressure, *P/F*, P_a_O_2_/FiO_2_ ratio; *P*_*v*_*CO*_*2*_ Partial pressure of carbon dioxide in venous blood, *Qt *Cardiac output, *SV *Stroke volume

The BGA shows low arterial oxygen levels (Table 4). The patient is hemodynamically stable without vasopressor support. The venous BGA shows a high ${S}_{{vO}_{2}}$: 86.7%.

To estimate the severity of the respiratory failure, the pulmonary shunt is computed and results 0.8 (80%). The arterial oxygen content is the same of the first two cases despite the presence of a much higher pulmonary shunt. This is due to the extracorporeal oxygenation of venous blood by V-V ECMO. The shunted venous blood is thus high in oxygen content and does result in severe desaturation (Fig. 4C). Of note, a shunt of 0.8 without ECMO is incompatible with life [9].

To measure the pulmonary shunt during VV-ECMO, patient global ventilation (membrane and natural lung) with the same oxygen fraction is preferred [32]. The FiO_2_ change on the ventilator and on the membrane lung is set to obtain a more consistent shunt measurement especially during repeated procedures over time. [33]. This could involve resetting the FiO_2_ on both the ventilator and the sweep gas. An adequate time after FiO_2_, changes must be allowed to get the new steady state before the shunt computation.

The minute ventilation on the natural lung and the gas flow on the membrane lung do not influence pulmonary shunt measurement. The use of high inspired oxygen concentrations is considered to estimate “true shunt” fraction for inhomogeneous alveoli, because high FiO_2_ can blunt the effect of hypoxic vasoconstriction. Intrapulmonary shunt will be always mildly overestimated because of a minimal constant contribution of a physiologic shunt (right to left shunt of the Thebesian and bronchial circulation) [34]. To be consistent and to compare the degree of shunts within the same patient, it is possible to use FiO_2_ at 1.0 [35].

After the intricate considerations surrounding pulmonary shunt measurements in V-V ECMO, it is essential to delve into the dynamics of oxygenation during venoarterial (V-A) ECMO, shedding light on the complex interplay between extracorporeal and native circulation.

During V-A ECMO, the oxygen content in the patient’s arterial blood is a combination of blood from two sources: the extracorporeal circuit and the left ventricle (i.e., the blood passing through the lungs). Consequently, in femoro-femoral V-A ECMO, oxygenated blood from the extracorporeal circuit moves from the femoral artery to the aorta, where it meets blood ejected from the left ventricle. The oxygenation of the latter depends entirely on the native pulmonary function. The precise location of this mixing point hinges on the relationship between extracorporeal blood flow and native cardiac output: the lower the cardiac output, the closer the convergence to the aortic root. This explains the critical role of measuring arterial ${P}_{{O}_{2}}$ from the right radial artery in V-A ECMO monitoring. While blood oxygenation from the ECMO circuit can be directly sampled at the ECMO outlet, the right radial artery, being closest to the heart and furthest from the ECMO oxygenator, is significantly influenced by the native lung function, particularly in the presence of a low cardiac output [36].

In cases of severely impaired native heart function, often observed in the initial phase of a V-A ECMO run, extracorporeal oxygenation may impact the O_2_ content of blood from all supra-aortic roots, especially on the left side. Consequently, patients frequently exhibit a higher arterial ${P}_{{O}_{2}}$, reflecting the elevated ${P}_{{O}_{2}}$ level of blood exiting from the oxygenator. Due to this dynamic, the oxygenation of blood sampled from the right or left radial artery may not be directly comparable. Therefore, in the presence of a pulmonary disease such as pneumonia or ARDS, shunt may be hardly assessed because arterial ${P}_{{O}_{2}}$ level depends on the arterial ECMO oxygenation and the degree of native cardiac output blood mixing. For this reason, calculating the shunt in V-A ECMO patient, even if with sufficient cardiac output, remains a potentially misleading issue.

## Limitations

Pulmonary shunt computation represents a more valuable way to assess the respiratory failure severity as compared to the simple analysis of oxygenation such as PaO_2_/FiO_2_ and arterial oxygen saturation. However, it is worth to underline that the shunt computational model is founded on two assumptions. First, being the capillary oxygen content not measurable, its value is expressed as equal to the oxygen content contained in the alveoli. Second, assuming the capillary oxygen content equal to the alveolar oxygen content, it implies to consider that the alveolar-capillary membrane is perfectly efficient. In other words, the entire shunt computation is conceived considering that all the oxygen delivered to the alveoli passes completely through the alveolar-capillary membrane, an assumption potentially different from the reality of the gas exchange. Furthermore, the shunt computation requires three different BGAs and a complex calculation which needs time and resources.

## Future directions

As the shunt estimation is clinically relevant and can provide key information on the severity of the lung injury of our critically ill patients with respiratory failure, at bedside, future areas of research in this field may be considered such as follows:

- Exploring less invasive methods, such as midline catheters, which have shown promising levels of accuracy comparable to more invasive options like Swan-Ganz or central venous catheters [37]. These modalities could be more extensively used in the future for blood sampling and shunt computation. Knowledge of the sampling sites allows clinicians to properly interpret the values obtained.
- Incorporating shunt assessment and its changes over time due to treatment into prognostic algorithms (e.g., machine learning) in the future, along with other parameters of gas exchange (e.g., PaO_2_/FiO_2_) and mechanical ventilation (e.g., driving pressure) to predict prognosis or outcomes.
- Considering the addition of shunt computation as a routine assessment of lung injury severity [18] during the routine clinical practice, when addressing certain, defined syndromes, such as ARDS.

## Conclusions

- Pulmonary shunt is a physiological variable that reflects the severity of respiratory failure.
- The computation of pulmonary shunt involves the measurement of O_2_ content at three sites: venous (obtained by a SG catheter sample or — with certain limitations — through a CVC), capillary (estimated based on the alveolar gas equation), and arterial (through an arterial catheter).
- Pulmonary shunt analysis is particularly valuable in clinical conditions where the analysis of BGA is biased by a significant alteration of venous O_2_ content, such as low-cardiac output conditions or V-V ECMO support.
- This review illustrates to future clinicians how to accurately measure and correctly interpret the shunt equation and how to evaluate different case scenarios by changing physiological variables by using an online shunt simulator.

## Data Availability

Not applicable.

## Abbreviations

ARDS: Acute respiratory distress syndrome
BGA: Blood gas analysis
C_*art*_: Arterial O_2_ content
C_*cap*_: Capillary O_2_ content
COHb: Carboxyhemoglobin
CT: Computed tomography
C_*ven*_: Venous O_2_ content
EELV: End-expiratory lung volume
FiO_2_: Fraction of inspired oxygen
FRC: Functional residual capacity
HbO_2_: Hemoglobin oxygen saturation in arterial blood
HR: Heart rate
MetHb: Methemoglobin
ML: Membrane lung
NL: Natural lung
PA: Arterial blood pressure
P_a_CO_2_: Partial pressure of carbon dioxide in arterial blood
P_a_O_2_: Partial pressure of oxygen in arterial blood
P_atm_: Atmospheric pressure
P_cap_O_2_: Partial pressure of oxygen in capillaries blood
PEEP: Positive end-expiratory pressure
P_H2O_: Vapor pressure
P/F: P_a_O_2_/FiO_2_ ratio
P_v_CO_2_: Partial pressure of carbon dioxide in venous blood
P_v_O_2_: Partial pressure of oxygen in venous blood
Qt: Cardiac output
Qs: Pulmonary shunt
RQ: Respiratory quotient
S_a_O_2_: Arterial O_2_ saturation
S_cap_O_2_: Capillaries O_2_ saturation
S_cv_O_2_: Central venous O_2_ saturation
SV: Stroke volume
S_v_O_2_: Mixed venous O_2_ saturation
V-A ECMO: Venoarterial extracorporeal membrane oxygenation
V/Q: Ventilation-to-perfusion ratio
V-V ECMO: Veno-venous extracorporeal membrane oxygenation
